# Evaluating the impact of added greenery on perceived factors of an urban environment in virtual reality

**DOI:** 10.1371/journal.pone.0316195

**Published:** 2025-02-06

**Authors:** Ron Bar-Ad, Markel Vigo, Geoffrey Caruso, Qudamah Quboa, Nuno Pinto

**Affiliations:** 1 School of Social Sciences, University of Manchester, Manchester, United Kingdom; 2 Department of Computer Science, University of Manchester, Manchester, United Kingdom; 3 Luxembourg Institute of Socio-Economic Research, Esch-sur-Alzette, Luxembourg; 4 Alliance Manchester Business School, University of Manchester, Manchester, United Kingdom; 5 School of Environment, Education, and Development, University of Manchester, Manchester, United Kingdom; University of Antwerp, BELGIUM

## Abstract

The wellbeing effects of urban greenspace are well established, but may be more attributable to pedestrians’ perceptions than objective levels of greenery. An immersive virtual environment was designed with three levels of roadside greenery: no trees, 200 trees, and 400 trees. Participants were asked to rate each for several perceived and objective factors, and gave their years lived experience in urban, rural, and suburban environments. Trees impacted perceptions of beauty and greenness, and, slightly, building heights. Controlling for urban experience significantly lessened the impact of trees, but showed that perceived greenness had higher significant correlations to all other outcomes.

## Introduction

As more and more of the world’s population moves to urban area [1], and with growing evidence of the connections between urban environments and poor wellbeing [2–8], research is being undertaken on the elements of urban environments thought to have the most impact—in particular, green space. The idea that urban green space can be strongly correlated to resident activity and wellbeing [9] has generated an interest in the efficacy of urban greenness as a tool for the promotion of physical activity and public health [9–25].

However, some papers cast doubt on these findings, suggesting it is the perception of greenness that affects the wellbeing of residence more than the objective measure of greenness does in itself (see [26–28]). While research has posited an impact on perceptions of urban spaces from personal background [29], and from community attributes [30–34], there is little research verifying the impact of personal experience on these perceptions [33,34].

To evaluate the perceptions of urban space, the use of Immersive Virtual Environments (IVEs) has gained popularity in recent years (see [35–38]). The use of virtual reality gives researchers the benefit of both the closest possible sense of presence to really being somewhere, and the highest possible level of control over the environment [35,39–44].

However, if data is to be collected on participants in an IVE, it should not interrupt their immersion or disrupt their sense of presence. One way to combat this is to rely on physiological and discrete-choice data [36,45,46], another is the use of in-VR questionnaires (IVQs), which do not require the user to exit the virtual environment [47,48].

Three research gaps can therefore be identified: one in the implementation of ecologically valid IVEs; one in the effect of urban greenness on perceptions of space; and one in the confounding role of personal background in the perceptions of urban greenness.

This article aims to address these gaps, by answering three fundamental research questions.

- How can immersive virtual environments be utilised in user-centred urban environment research?
- How does the inclusion of greenery impact pedestrian perceptions of urban space?
- How does lived experience of urban areas affect said perceptions?

To address these questions, we present an experiment wherein participants in an IVE are shown a procedurally generated city street with and without roadside trees, and are asked to rate each version for beauty, safety, density, greenness, building height, and road width.

The Literature Review section will pertain to perceptions of urban greenspace and its link to wellbeing, as well as the use of virtual reality in researching this field. The Review of Existing Approaches section will further discuss existing uses of IVEs to research perceptions of the urban environment. The Methods section will cover this study’s approach and design, Findings will cover the results of our experiment, and Discussion and Conclusions will give a final discussion and conclusion.

## Literature review

This review will cover the effects of urban green space and its perception in subsection Perceptions of Urban Green Space, and the use and validity of IVEs in subsection Immersive Virtual Environments.

### Perceptions of urban green space

Past approaches to the examination of the effects of urban green space rely on different measures of greenery, from park proximity to Geographic Information System (GIS) models of land use to presence of road-side vegetation and many more [49]. Nevertheless, the literature generally suggests a link between resident wellbeing and greenery, in one form or another [50]. Park proximity is an established predictor of physical activity, which is highly related to physical health [20,25,51–55]. Greenery and green spaces are strongly connected to psychological wellbeing [13,19,21,23,24,56–60], green spaces are associated with social connections [16,56], and other studies show strong connections between urban greenspace and morbidity [61], stroke mortality risk [62], and general health [12,15,18,63].

However, [28] casts a doubt on these findings, showing that the perception of greenness on its own improves mental health and encourages walking, which improves physical health. [26] supports this in finding that perceived friendliness of an urban environment is directly connected to self-rated health. [27] similarly showed that people tend to be happier in more scenic locations, scenic-ness being a perceived and subjective quality. Further, [64]‘s concept of ‘just green enough’ urban planning, that attempts to help working class areas without opening them up to gentrification, finds a balance between as much of the health benefits of green space as possible with as little possible effect on perceived property value (also see [63]‘s discussion of the necessity of ‘just green enough’ design).

This is supported by studies more adjacent to urban greenery. Several studies have shown an apparent mismatch in the variations of urban biodiversity and its perception [65,66] showed that it’s the mixed land use that most impacts land prices, rather than the presence of greenery itself. [67] found that the well-being of greenspace visitors was connected to their perception of the space’s species richness, but found no consistent pattern with actual, measured species richness. [68] found that, while visitors expressed a preference for species richness, they did not notice increased flower diversity when it was present.

The perception is not only affected by present environment, but also by the individual and their background. [65] found that highly urban-oriented and highly nature-oriented individuals perceived more urban greenery than their less-oriented counterparts. [69,70] show that cultural and personal attitudes can affect an individual’s perception of an urban environment, [71] found that childhood use of greenspace is an important predictor of adult use of greenspace, [72] finds that children raised in city environments are more predisposed to have a positive view of urban greenspace, and [73] finds that childhood perceptions of nature can predict some adult greenspace visitation patterns.

This is underpinned by [74]‘s finding that visitation of greenspace reflects its perception, specifically its perceived safety.

The idea that perception of the urban area may be as key to wellbeing as its objective qualities is not specific to urban green space. [75–77] all described the different ways urban density can be perceived and evaluated differently by people from different cultures and countries, finding that people can ‘read’ an environment as ‘dense’ or ‘not dense’ irrespective of (or partly independently of) the actual number of people per unit area. [29] theorised a notable number of personal and environmental factors that may influence an individual’s perceptions of an urban environment; and more recently [78] found that, above a certain threshold, acceptance of denser areas was mostly affected by participant perceptions (for example their perception of the politics of urban densification). [38]‘s finding, that the effects of increased urban density can to some extent be mitigated by mixed land use and urban greenery, would suggest that these fields are strongly connected. It is not only that density and greenery are both values that can be interpreted separately in their objective and perceived forms, but that both form part of the larger impact of the perception of urban environments.

These studies together would suggest that it is not simply the objective measure of greenness in an urban space that improves the wellbeing of residents, but the perception the residents have of the space that makes the difference. This would imply that the elements that influence an inhabitant’s perception of that greenness can be controlled to improve their wellbeing, without necessarily changing the actual, objective greenness of the area.

### Immersive virtual environments

Considerable research has already been undertaken trying to understand the validity of VEs in general and IVEs specifically. [79] found that experiments showing an immersive 360-degree video on a head-mounted display can substitute field experiments, and show no significant difference in user self-reports. [80] found that pedestrian responses to an environment in immersive 360-videos are largely similar to those in the real world, and induce less distortion than still images. [81] found that perceptions of an environment did not differ between a field study at a real-life location and a lab study of an interactive IVE representation. Other studies found that participants in a virtual environment behaved similarly to real life, with a variety of approaches to the VE itself [82–86].

All these works together establish a pattern of consistency between in-VR behaviour and in-real-life behaviour, creating a foundation of ecological validity from which VR studies can continue. IVEs specifically are fully immersive, in that the user is perceptually surrounded by the virtual environment. This includes head-mounted displays (HMDs) and Cave Automatic Virtual Environment (CAVE) systems, which are both shown to be highly immersive [87], though CAVEs require stereoscopic glasses and large spaces. They can show results even closer to reality than traditional choice experiments or 360-degree still images [88,89], and can provide more realistic feelings and more useful data [90,91] found that, for landscape planning, “the immersion, motion, and sound offered by virtual worlds may greatly extend the ecological validity of environmental representations and allow for deeper and more meaningful study of the effects of the real world on human experience.” (pp. 139).

The ecological validity of IVEs can be assessed through subjective response surveys, cognitive performance tests, and physiological responses [46], usually on the metrics of immersion and presence [36]. Immersion can be expressed as the extent to which the IVE and the hardware delivering it create an “inclusive, extensive, surrounding and vivid illusion of reality” [92] (pp. 606). Presence is considered to be a sense of being in the virtual environment, despite not physically being there [36,93–95]. The higher the measures of presence and immersion, the more ecologically valid an IVE is considered to be [82,96].

Presence and immersion can be increased through higher levels of realism [48,82,96,97], which can be expressed through photo-realism [98], soundscape realism [99,100], or interactivity [92]. However, they can be interrupted by disrupting the participant’s connection to the virtual environment, for example by removing their headset [47,48] or even using teleportation locomotion (a method of traversing the virtual environment by pointing at a location and appearing there) [101].

As noted above, ways to avoid breaks in presence and immersion are relying on physiological and discrete-choice data [36,45,46] and using in-VR questionnaires (IVQs), which do not require the user to exit the virtual environment [47,48].

### Review of existing approaches

VR has long been used in urban planning and architecture research, often with the intention of evaluating the simulation itself as a research method [102].

An example of IVEs specifically is [36], who created a highly immersive environment by expertly combining Unity store assets in the style of a typical urban area, rather than replicating a specific location or relying on existing models. They measured both physiological metrics such as electrodermal activity and heartrate, and user self-reports in the form of IVQs. They tracked user position at all times and gaze fixation on two virtual objects. The IVE was experienced through a HMD and interacted with through a walking controller (a VR treadmill).

Another IVE study to consider is [38], who used a large concave screen with a 75-degree field of view to project short videos simulating a walk down a virtual replica of a real street. This approach is similar to the CAVE system, in which a set of screens, projectors and motion-detection cameras are set up within a designated room [36], the key difference appearing to be that CAVE systems are generally interactive. In terms of immersion, there does not seem to be a significant difference between CAVE systems and HMD systems [90,103,104].

Both [36,38] used hand-made virtual environments, one with custom assets chosen and assembled to create the effect of a familiar urban setting, and one using repeated patterns to replicate an existing street layout in different configurations. This level of control allows the potential for more immersive environments, for example by using high-quality assets or expert environment designers. Both these approaches require the experience and time to design a virtual environment. [105] lists the steep learning curve and experience necessity of using VE development tools as one of the two most significant drawbacks of VR research, alongside the large space requirement.

In terms of hardware, [38] has the larger space cost by requiring a room for the 2.4x7.5-metre screen, but can include participants in groups in order to save on time. [36] only needs enough room for the walking controller, but can only immerse one participant at a time. This introduces a sort of time-cost in terms of sample participation—a larger sample has a direct and linear relationship to the time required for the data collection in both cases, but one is decidedly lower than the other.

The equipment required for both approaches is expensive, one using a large screen and two projectors and another using a walking controller and HMD. [36] also utilises physiological sensors, one for EDA, cardiovascular activity and skin temperature, and another for gait analysis. [38] also has the upfront expense of the venue required for screenings.

We can therefore identify four metrics by which we can compare the two approaches:

- **Ease of Development**—the experience required and difficulty associated with the development of the VE and integration of the hardware;
- **Money-Cost of Development**—the financial expense of the hardware and software used;
- **Time-Cost of Experiment**—the number of participants who can be immersed in the VE at a time;
- **Level of Immersion**—the amount the participant can really feel they are physically present in the VE.

*Level of Immersion*, as discussed in the Literature Review, can be measured by [92]‘s objective framework, by physiological and psychological measures of presence, or by qualitative analysis. [92] identifies six objective elements of an IVE that can measure its impact on immersion: *Inclusive* (the extent to which physical reality is shut out), *extensive* (the range of sensory modalities accommodated), *surrounding* (extent of field of view—panoramic vs narrow), *vivid* (resolution, fidelity, variety of energy simulated, richness and information content), *matching* (accuracy of system responsiveness to user interaction, e.g. turning head should turn perspective in a 1:1 relationship), and *plot* (extent to which VE presents a narrative of internal events distinct from those happening in the real world). For example, a setup like [36]‘s might score highly on inclusivity, extensiveness, and surrounding thanks to the use of a HMD, and vividness, matching, and plot thanks to a hand-made virtual environment and high-spec hardware.

*Time-Cost of Experiment* is a simple evaluation of the number of participants able to engage with the VE at a time. This may also include the amount of time required to train participants in the use of the hardware, to monitor them to ensure their safety, and even simply the average length of their participation.

*Money-Cost of Development* is the financial cost of every piece of equipment, of the space they occupy, for the time they are used. This will be affected of course by the Time-Cost of Experiment—prolonged use of the equipment may cost more on loan, or simply create wear that will require maintenance—and by Ease of Development—the more time is taken to develop the VE the more the hardware must be used or loaned or worn in testing.

Finally, *Ease of Development* may be the most subjective of these measures. Does one need to consider the experience of the researcher as they begin development as part of their training? Or should one only consider the time from the start of development to the end? Do different tools have different learning curves in VE design? Unfortunately, these questions, to the best of our knowledge, are yet to be answered.

We have then identified four fields for improvement when developing our own IVE approach to examining perceived qualities of urban environments. We must maximise Ease of Development and Level of Immersion, while minimising Money-Cost of Development and Time-Cost of Experiment.

## Methods

### Ethics

This research was approved by the University of Manchester School of Social Science Ethics Committee under review reference 2022-13711-24657.

### Sampling

This study employed a convenience sampling method, with a sample of n = 34 participants, recruited between 10/10/2022 and 19/10/2022. Informed written consent was obtained for all participants. No demographic data was collected aside from their experience living in urban, suburban and rural environments, measured by number of years. Many more participants had lived in urban areas for most of their lives, with 18 having more than 20 years of experience living in urban areas, compared to just 1 participant with more than 20 years in suburban areas and no participants at all with more than 20 years in rural areas.

**Fig 1:**
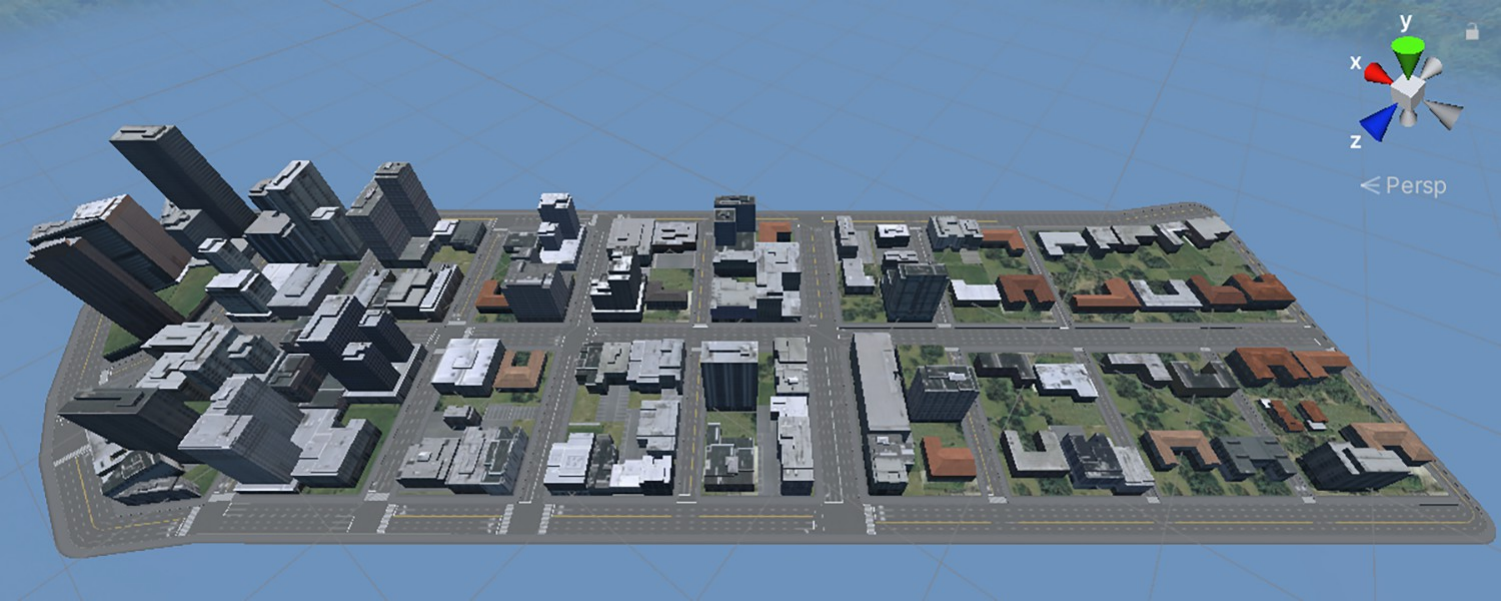
Sample lived experience per environment.

Fig 1 shows a breakdown of the demographic results. No other information was collected on the participants.

### VR setup

Using the metrics outlined in the above section, an IVE was designed to best balance the four requirements specified. To maximise Ease of Development, we did not replicate a real-world location, nor did we hand-design a virtual location to resemble reality. Instead, we used a 3D street model procedurally generated by ESRI’s ArcGIS CityEngine [106]. This required minimal learning to use in its basic settings, though future iterations can be improved alongside understanding of the tool.

To minimise Money-Cost, the hardware used was a Meta Quest 2, one of the cheaper high-functioning HMD sets. It was chosen for its high specifications (1832x1920 pixel resolution per eye, 120Hz refresh rate) and its ubiquity, at time of writing making up 38% of VR devices used on Steam [107]. This decision was made with reproducibility in mind, as the most ubiquitous headset may be the easiest for future researchers to access. The hardware was integrated into the VE using Microsoft’s Mixed Reality ToolKit [108], a system-independent package that would theoretically allow the program to run on any VR hardware.

A questionnaire was designed for participants to answer as they walked through the environment, and to maximise Level of Immersion it was taken within the VE, so that no HMD-exiting break in presence would occur while answering questions.

### The virtual environment

**Fig 2:**
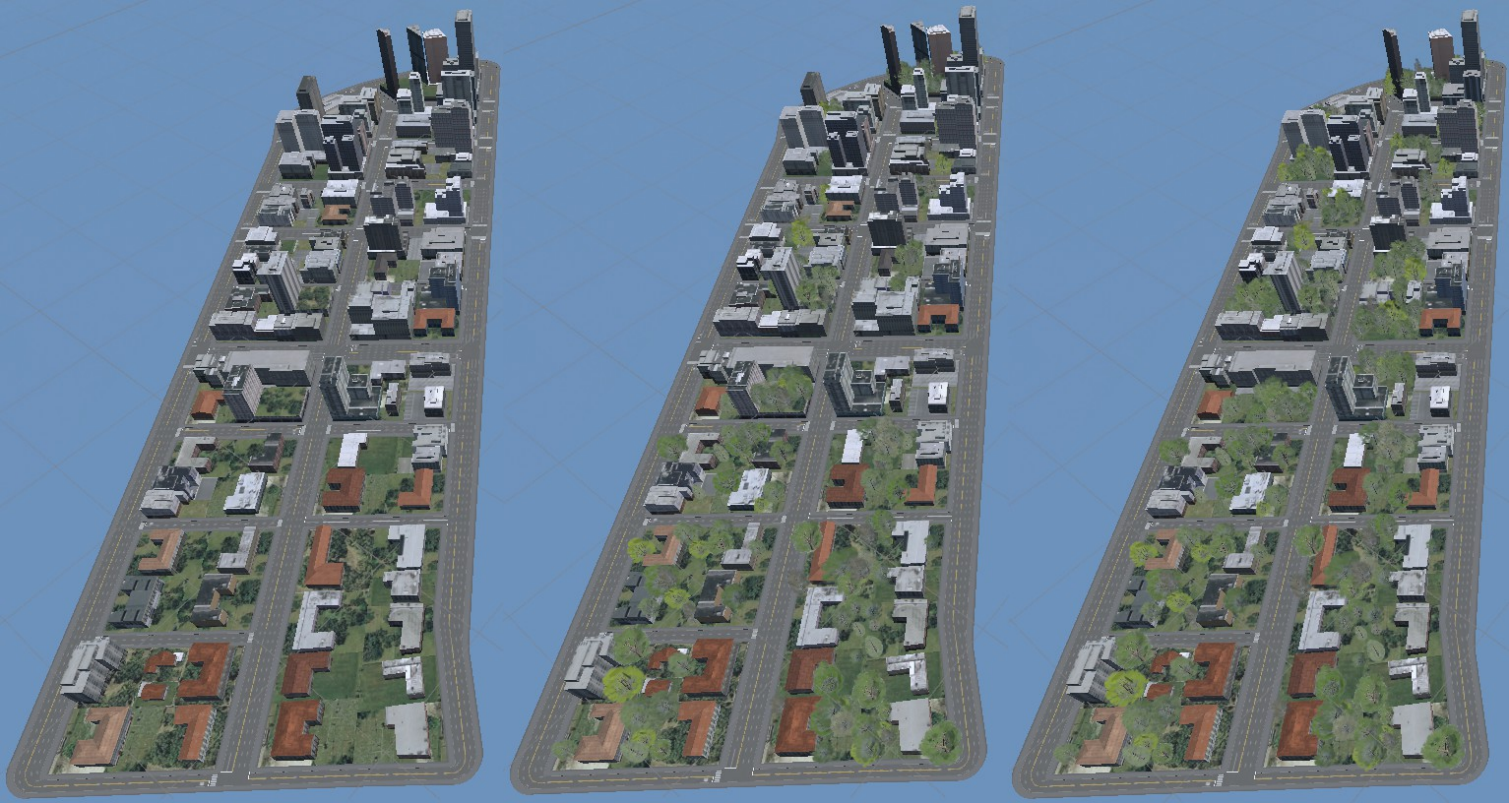
A bird’s-eye-view of the VE with no trees.

The VE, imported to Unity from CityEngine, consisted of a handful of city blocks abreast a main road with increasing urban density, such that buildings at one end of the road were much taller than those at the other end. A bird’s eye view can be seen in Fig 2.

CityEngine generated the city these blocks were part of through a procedural generation process based on the street network of Berlin, then generated the building outlines for each block and extruded buildings to a height and shape based on their distance from the centre of the city. The program then gives each building a facade texture, and projects satellite imagery onto the ground to represent grass or vegetation.

**Fig 3:**
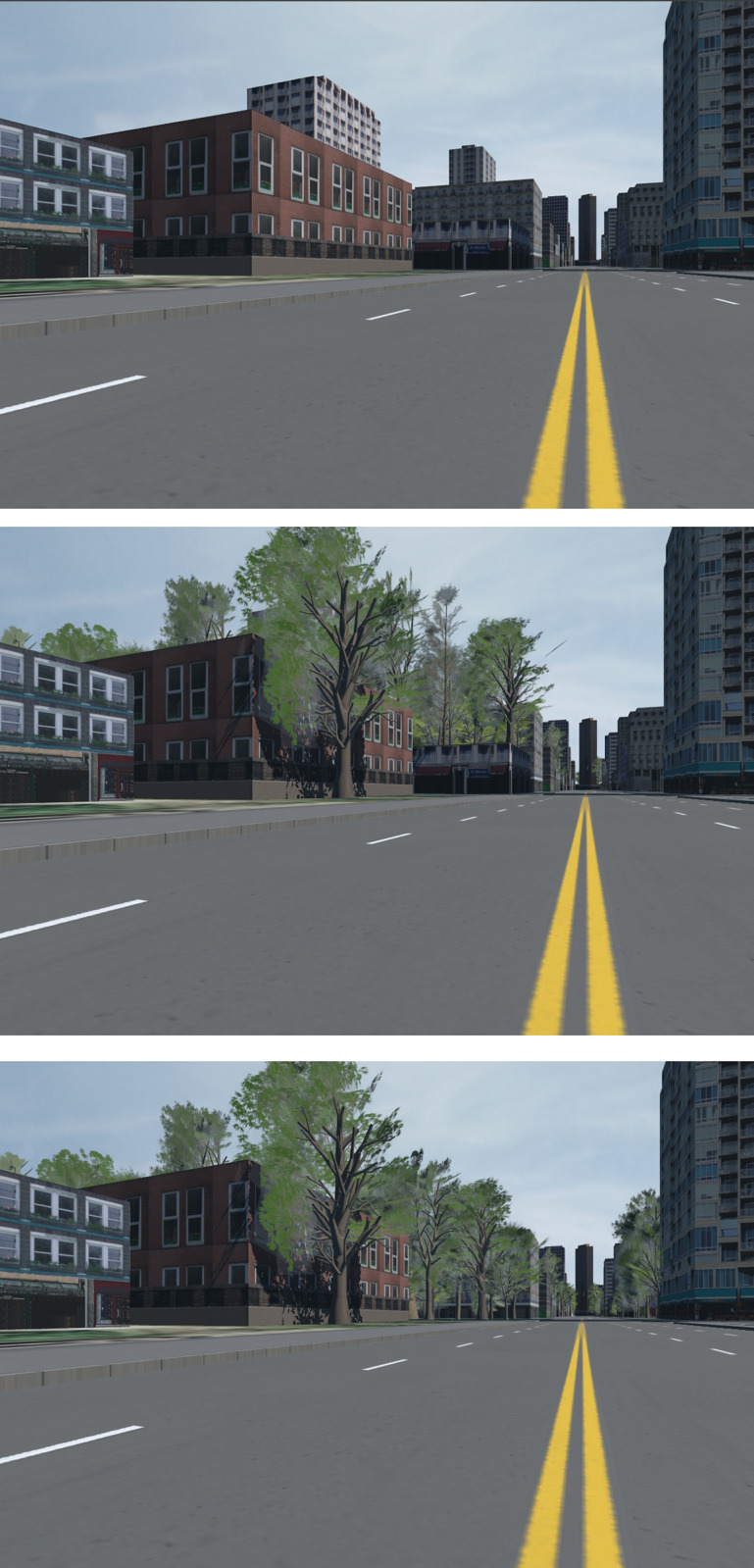
Environments (from left to right) *NoTrees*, *SomeTrees and MoreTrees*, side by side.

**Fig 4:**
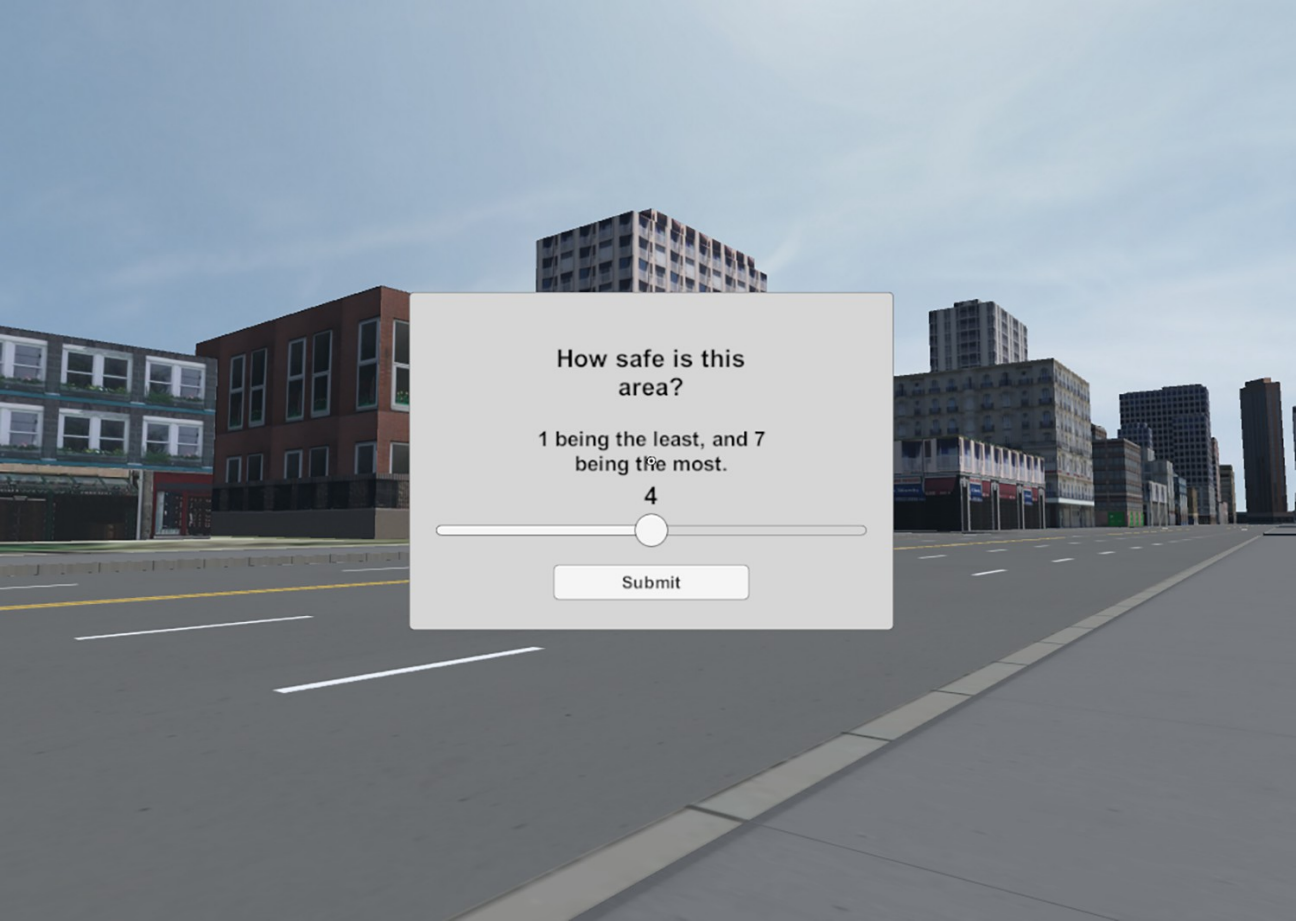
Environments (from top to bottom) *NoTrees*, *SomeTrees and MoreTrees*, from a street-level perspective.

Three such environments were made, *NoTrees* with no trees whatsoever (those seen in Fig 2 are in fact satellite projections and part of the grass), *SomeTrees* with trees as they would normally be generated by the program, and *MoreTrees* with some buildings replaced with groups of trees. The three environments can be seen side-by-side in Fig 3, and a ground-level view in Fig 4.

An asset count reports the number of trees in each environment, as seen in Table 1.

**Table 1:** Number of tree assets per environment.

Environment	Number of Trees
*NoTrees*	0
*SomeTrees*	232
*MoreTrees*	419

The realism of the system was potentially limited by three disadvantages: (1) Sound was not incorporated in this system; (2) the photorealism was limited by the assets imported from CityEngine; and (3) there were no moving or interactable objects outside of the questionnaire. These factors may affect *matching* and *plot* elements of the [92] framework, but points (1) and (2) may also have added effects on the findings that are outside the scope of this paper.

### Experiment design

The study itself was straight-forward. Participants would put on the headset with assistance from the researcher, then be instructed on how to use the interface to open a program. They would then open the in-built introduction program *First Steps*, which would walk them through the different buttons on their controllers and acclimatise them to the experience of being in VR. During this time the researcher would be monitoring them for signs of nausea or disorientation (e.g. sweating, imbalance, fidgeting with HMD) [104].

After the participant has had time to get used to the headset and the researcher was satisfied they were not at an obvious risk, they were instructed to open the study program and walk around to get a feel of the environment. Interaction outside of movement was limited to the slider and buttons of the questionnaire panel. When the participants pressed the grip of their controller to bring up the questionnaire, they could move the slider or press the buttons using a cursor based on motion control, and a button on the controller. Locomotion involved mixed methods: participants could physically walk about the room, up to the virtual boundary set up by the researcher to ensure their safety from obstacles and walls, or they could push a thumbstick on the Quest’s handheld controller to move in the direction they were facing. The former method is known as ‘real walking’ or ‘real locomotion’ and is considered the locomotion method with the highest interaction fidelity, which is associated with immersion [109]. The use of mixed locomotion is uncommon, but [110] found that it was preferred by participants in a musical composition context for its allowance of control. Participants could utilise both forms of motion to explore their surroundings however they were most comfortable, though the physical walking was much more limited in range.

The task participants were given was to consider the tallest buildings and the shortest buildings, the nicest areas and the least nice areas, and, when they were ready, press the button on their controller that brings up the questionnaire. They could take as long as they wanted to end the task and begin the questionnaire, though they were encouraged to begin if more than 10 minutes had passed.

Participants would then answer a questionnaire which asked them to rate the environment on a series of qualities, on a scale of 1 to 7. These qualities were safety, greenness, density, beauty, building height, and road width.

Once all questions were answered, participants were moved to the next environment (e.g. from *NoTrees* to *SomeTrees*) and the process would be repeated.

### Participant monitoring

Virtual experiments including navigation tasks with unsupervised samples, for example those performed remotely, have shown little difference in completion and correctness of answers [111] and performance [112]. In order to ensure participant safety while in VR, this experiment employed a semi-supervised approach, in which participants were observed externally but their in-VR behaviour was not monitored.

The researcher stayed in the room and continually observed participants for any signs of disorientation, if any were found the participant would be asked if they needed to sit down or take a break.

The researcher could not see what the participants were seeing, as the Quest 2’s ‘casting’ feature was found to create latency that would influence perceptions of the environment and introduce unwanted physical symptoms [113], but regularly checked in on participant progress. Each participant had up to 30 minutes total, including the tutorial, environments, and questionnaires. They were encouraged to leave the tutorial after 10 minutes if they showed no signs of disorientation and had not already asked to move on themselves. This left at most 20 minutes per participant for the experiment.

Participants had to trigger the questionnaire themselves, ending the task. Some participants employed a think aloud approach, informing the researcher of their progress in the task, some also chose to engage with the real walking locomotion method, both of these factors giving the researcher a clear start and end point for the tasks between the questionnaires.

### Questionnaire

**Fig 5:**
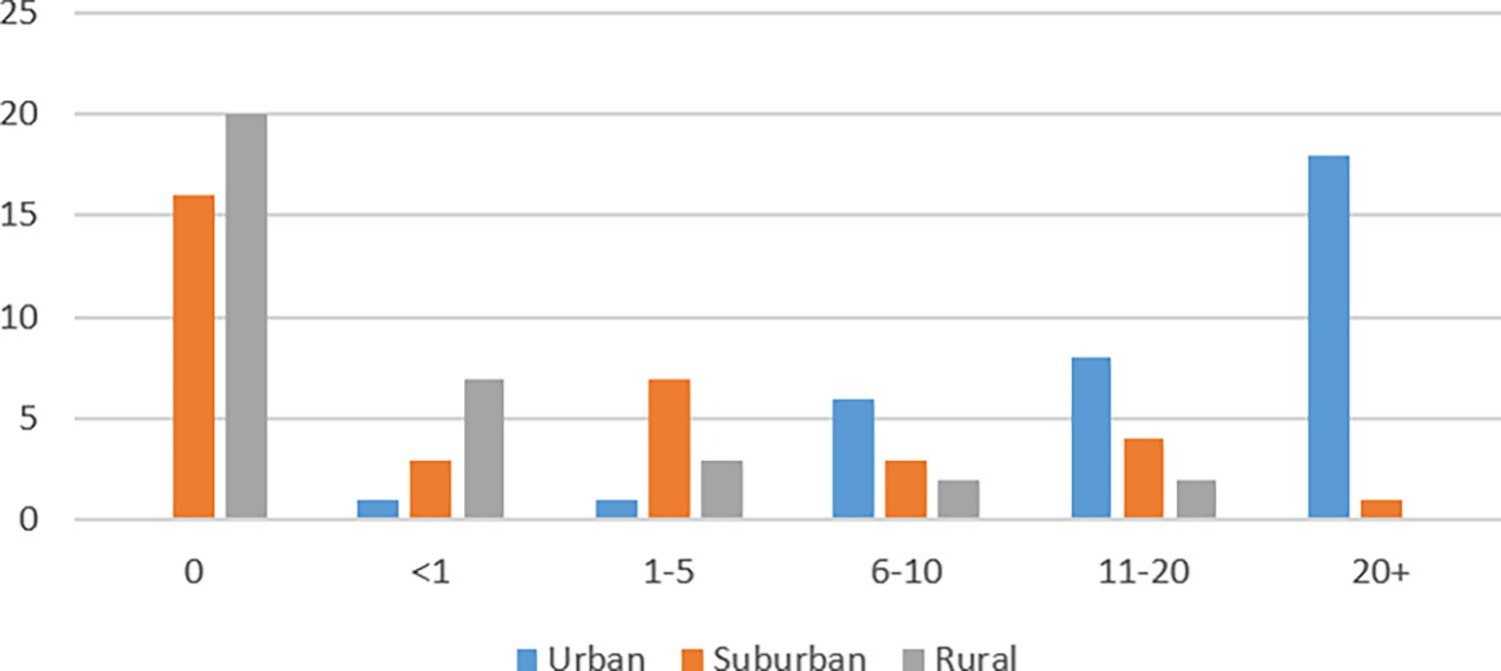
A view of the *NoTrees* environment with the questionnaire up.

The questionnaire contains six questions, each answerable on a scale of 1 to 7. Fig 5 shows the change in view when the questionnaire is triggered.

The questions were phrased plainly, as in Fig 5, they simply read “How beautiful is this area?”, “How tall are the buildings in this area?”, etc., followed by a reminder of the 1–7 scale.

The qualities assessed were chosen based on existing research of urban environment perception. Safety was chosen for its link to use of urban green space (see [19]). Beauty for its link to wellbeing [27]. Density for its association with wellbeing as well as its link to greenery [38]. Road width and building height to examine the effect of greenery on objective measures, but also for their associations with walkability and wellbeing (see [38,114,115]). Finally, greenery is included to compare the changes in ratings of greenness against the actual objective difference in number of trees.

### Pilot study

Before the experiment was carried out, a pilot study tested the feasibility and use of the approach with n = 11 participants. The study used an earlier version of the same program, the one difference was the questionnaire not yet being taken within the VE. Instead, the researcher gave participants the choice of either being read the questions out loud and answering out loud, or waiting to answer the questionnaire directly after they had seen each environment. Most participants chose the first option.

The pilot also asked participants to rate their experience living in urban, suburban, and rural areas on a 7-point scale from ‘None at all’ to ‘A great deal’, rather than reporting years lived.

The pilot found significant causal connections between the presence and number of trees and participants’ ratings of greenness, beauty, and, to a small extent, road width, but was limited by its small sample size and out-of-VR questionnaire.

### Findings

The outcomes of interest for this study were perceived safety, perceived beauty, perceived density, perceived greenness perceived building height, and perceived road width.

For a better idea on the structure of the data, a given participant’s responses can be seen in Table 2.

**Table 2:** The collected data on one participant.

Variable	Value
ParticipantID	13
Safety NoTrees	5
Safety SomeTrees	6
Safety MoreTrees	5
Density NoTrees	4
Density SomeTrees	5
Density MoreTrees	5
Beauty NoTrees	4
Beauty SomeTrees	6
Beauty MoreTrees	6
Greenness NoTrees	5
Greenness SomeTrees	7
Greenness MoreTrees	7
Building Height NoTrees	6
Building Height SomeTrees	3
Building Height MoreTrees	6
Road Width NoTrees	5
Road Width SomeTrees	5
Road Width MoreTrees	5
Lived Urban	Yes
Years Lived Urban	More than 20 years
Lived Suburban	No
Years Lived Suburban	0
Lived Rural	No
Years Lived Rural	0

**Fig 6:**
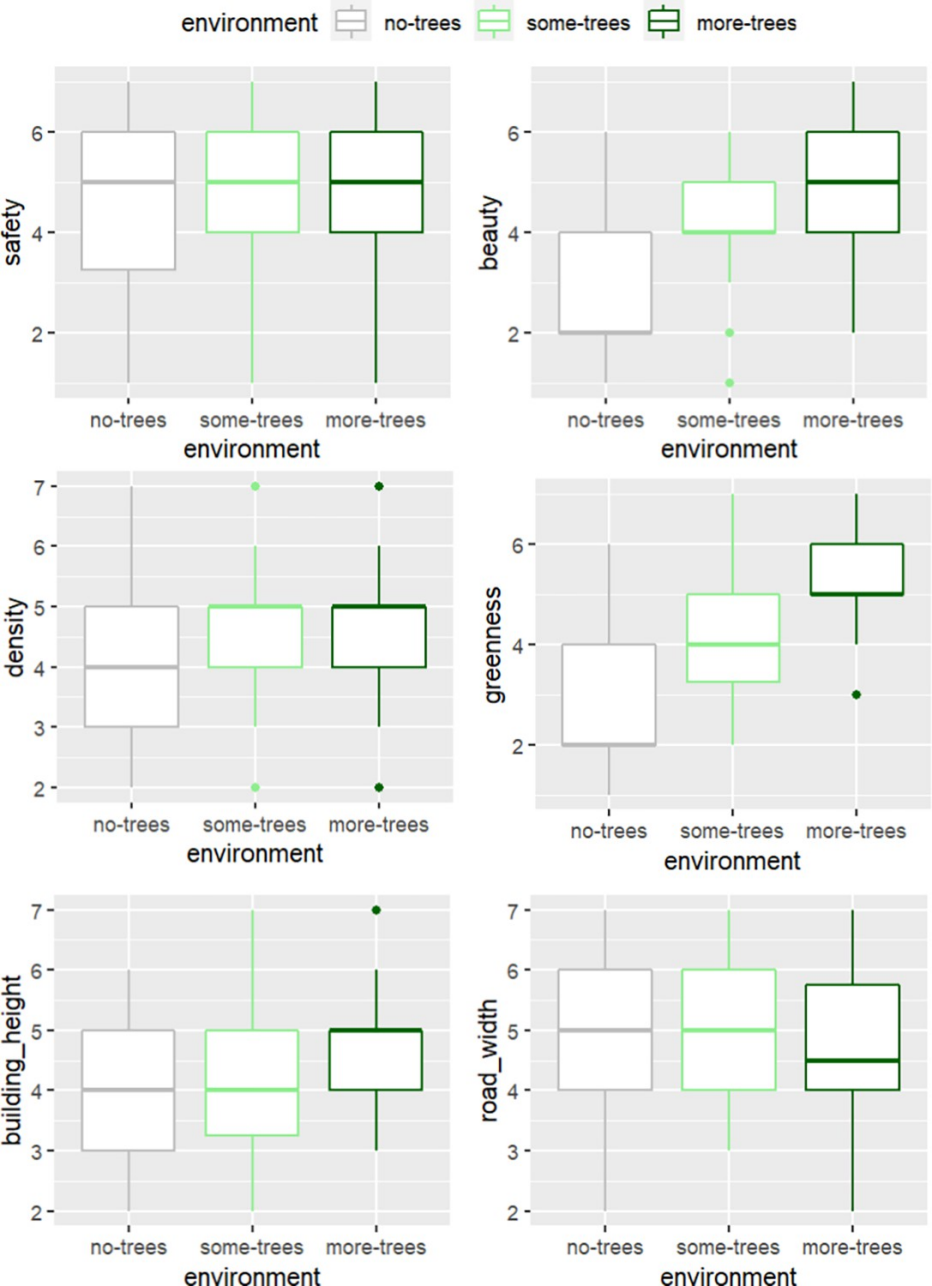
Boxplots showing the means and standard deviations of each outcome variable across the three environments.

Fig 6 shows the mean values and standard deviations for each outcome variable across the three environments.

An already visible trend seems to show that increasing the number of trees in an environment will increase ratings of perceived safety, beauty, and greenness and reduce perceptions of road width, but will also increase perceptions of density and building height. These trends are small for most variables, staying mostly within the 4–5 range of ratings, and showing that the *MoreTrees* environment had a somewhat more notable impact on the ratings than the *SomeTrees* environment. For perceived beauty and greenness, the boxplots show a large increase in ratings between *NoTrees* and *SomeTrees*. Ratings of beauty begin to diminish by *MoreTrees*, but greenness continues near-linearly.

A multivariate single analysis of variance model was fitted to determine the impact of the environment on all of these outcomes collectively, and univariate linear regression models were fitted to evaluate its effect on the outcomes individually.

Table 3 shows the results of the multivariate single analysis of variance model, which measures the contribution made by the environment on the variance of the outcome variables. A Pillai trace value closer to 1.0 indicates a larger effect of the variable on the model. Hotelling-Lawley and Roy’s Largest Root work similarly, with larger values indicating greater effect. Wilk’s Lambda is inverse, a value closer to 0.0 indicates a larger effect. These results together show that the number of trees has a significant impact on the outcome variables.

**Table 3:** MANOVA results.

Test	Result	p-Value
Pillai Trace	0.5856	2.542e-09
Wilk’s Lambda	0.44651	1.118e-11
Hotelling-Lawley Trace	1.2058	4.914e-14
Roy’s Largest Root	1.1771	3.242e-14

**Fig 7:**
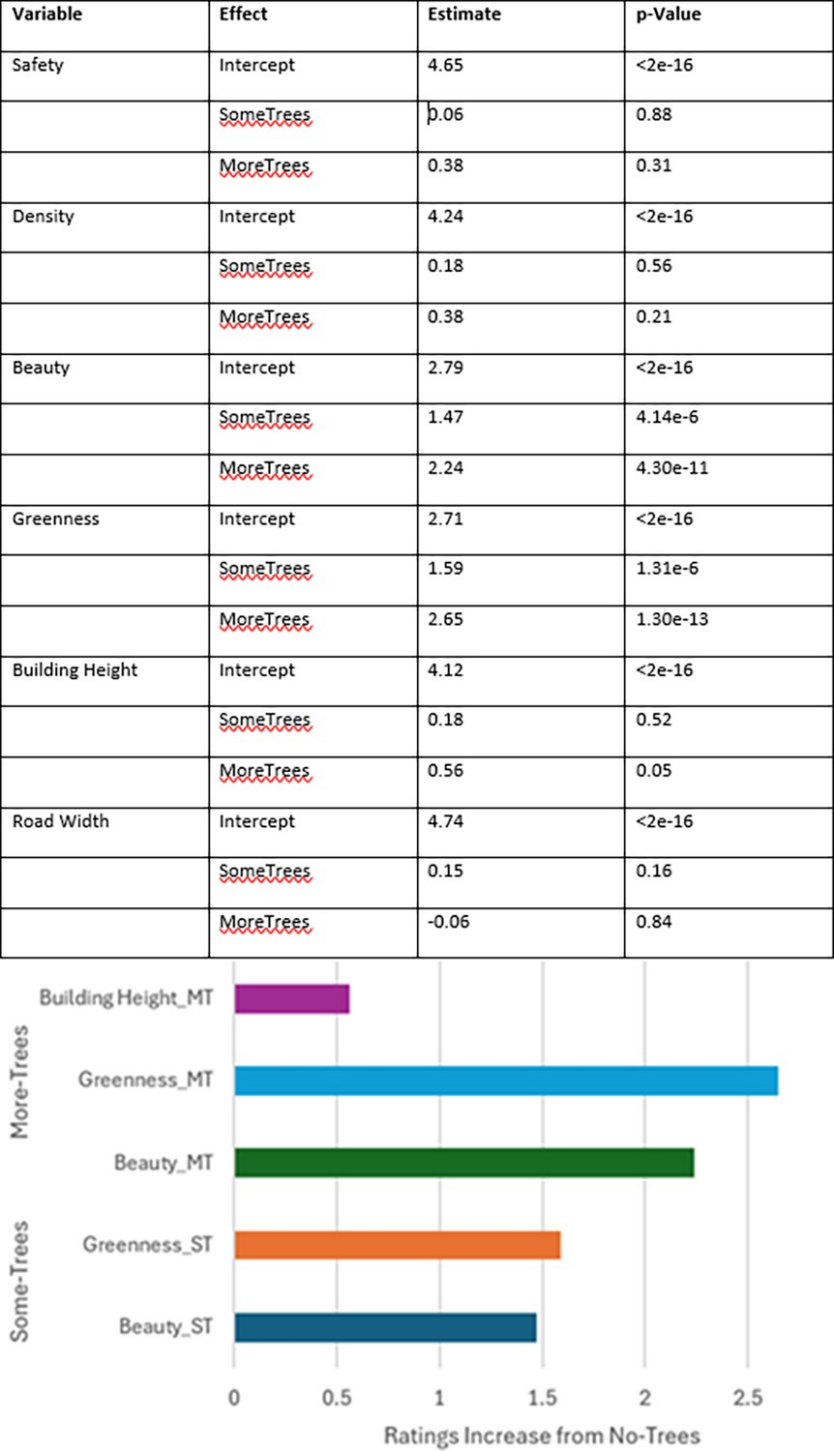
Detailed linear model results table (top) and significant findings of linear regression fitted to environment against all variables (bottom).

Each outcome was then fitted against the environment variable in a univariate linear regression. Fig 7 shows the ratings regardless of environment were generally above median for all outcomes, and that the absence of trees seemed to have a large impact on responses than the number of trees once they were present.

**Fig 8:**
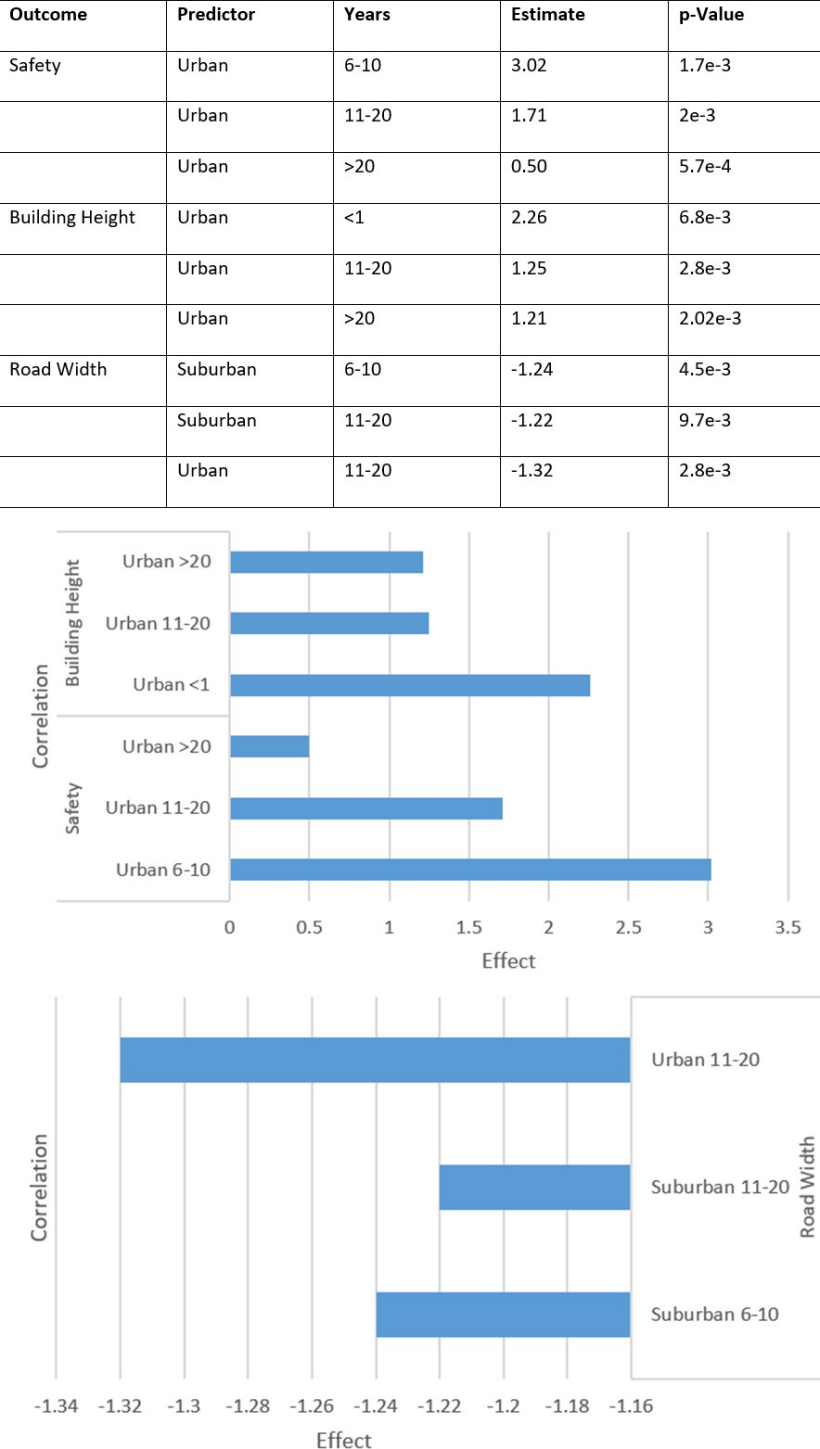
Detailed results of effects of background variables on perceptions (top) and visualisation of significant findings (bottom).

Focus should then shift to the lived experience variables, the participants’ years lived in urban, suburban, and rural environments. Linear regression models were fitted against the experience variable for each outcome. Fig 8 shows the significant findings with the largest coefficients from each model.

Many results did not have a p-value <0.05, likely reflecting the very small proportion of participant lived experience for suburban and rural areas.

The impact of number of trees when controlling for years lived in urban environments is considerably lessened for safety, density, and road width, so much so that they have no significant correlation between environment and outcome when controlling for lived experience.

**Fig 9:**
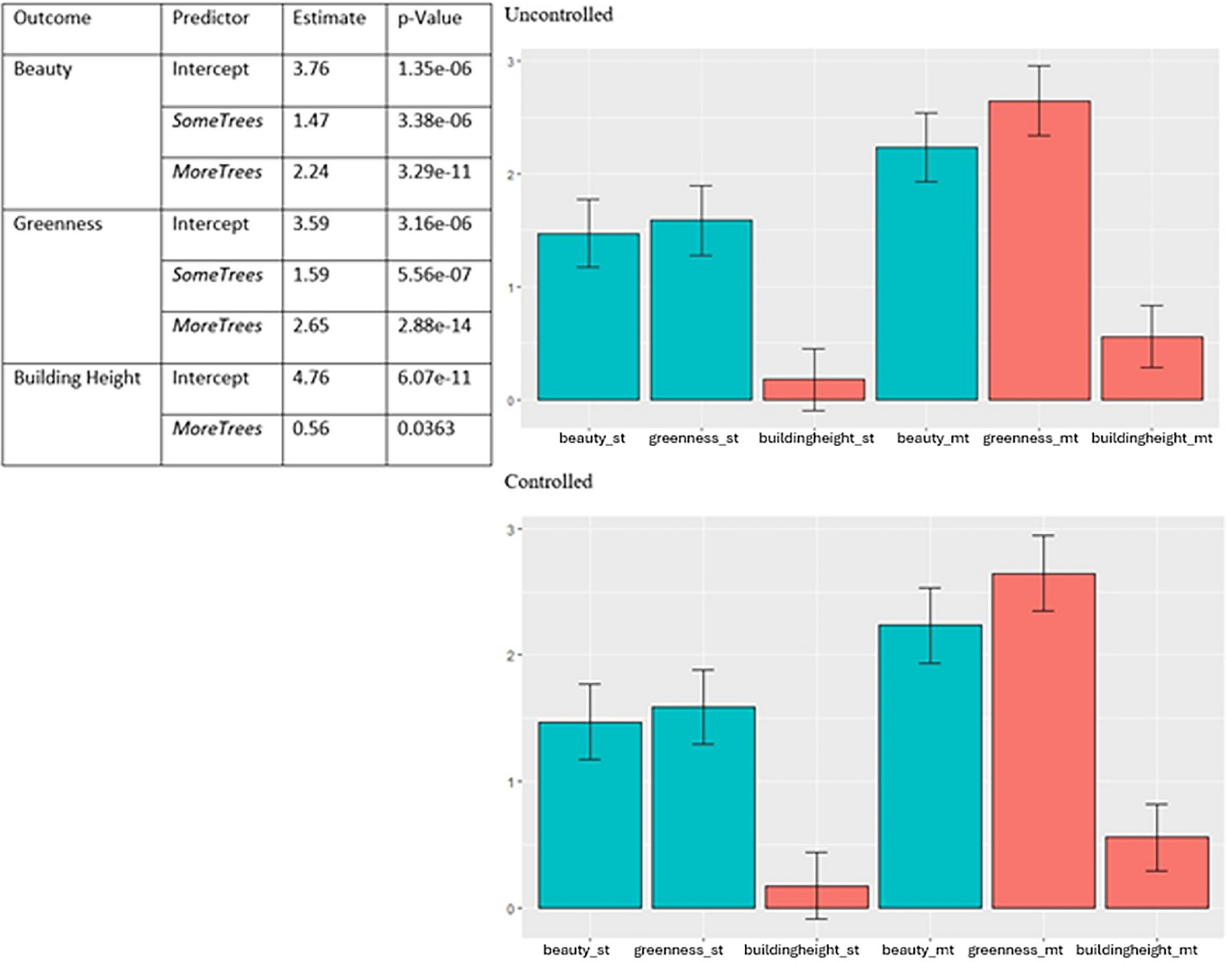
Side-by-side: Significant results of linear model controlled for years lived experience (right) and comparison to uncontrolled levels (left).

Building heights, beauty, and greenness, however, are significantly impacted by number of trees even when lived experience is controlled for. Fig 9 shows that significant findings from the initial linear model are unchanged when controlling for years lived in urban environments, with a difference in standard error as small as +-0.01.

However, when controlling for years lived and for environment, the relationship between perceived greenness and perceived qualities becomes one of predictor and outcome. Table 4 shows the impact of perceived greenness on perceived beauty, safety, and density.

**Table 4:** Impact of perceived greenness on outcome variables, from linear models controlling for years lived in urban areas.

Outcome	Estimate	p-Value
Safety	0.31	<0.001
Beauty	0.67	<0.001
Density	0.23	0.002

**Fig 10:**
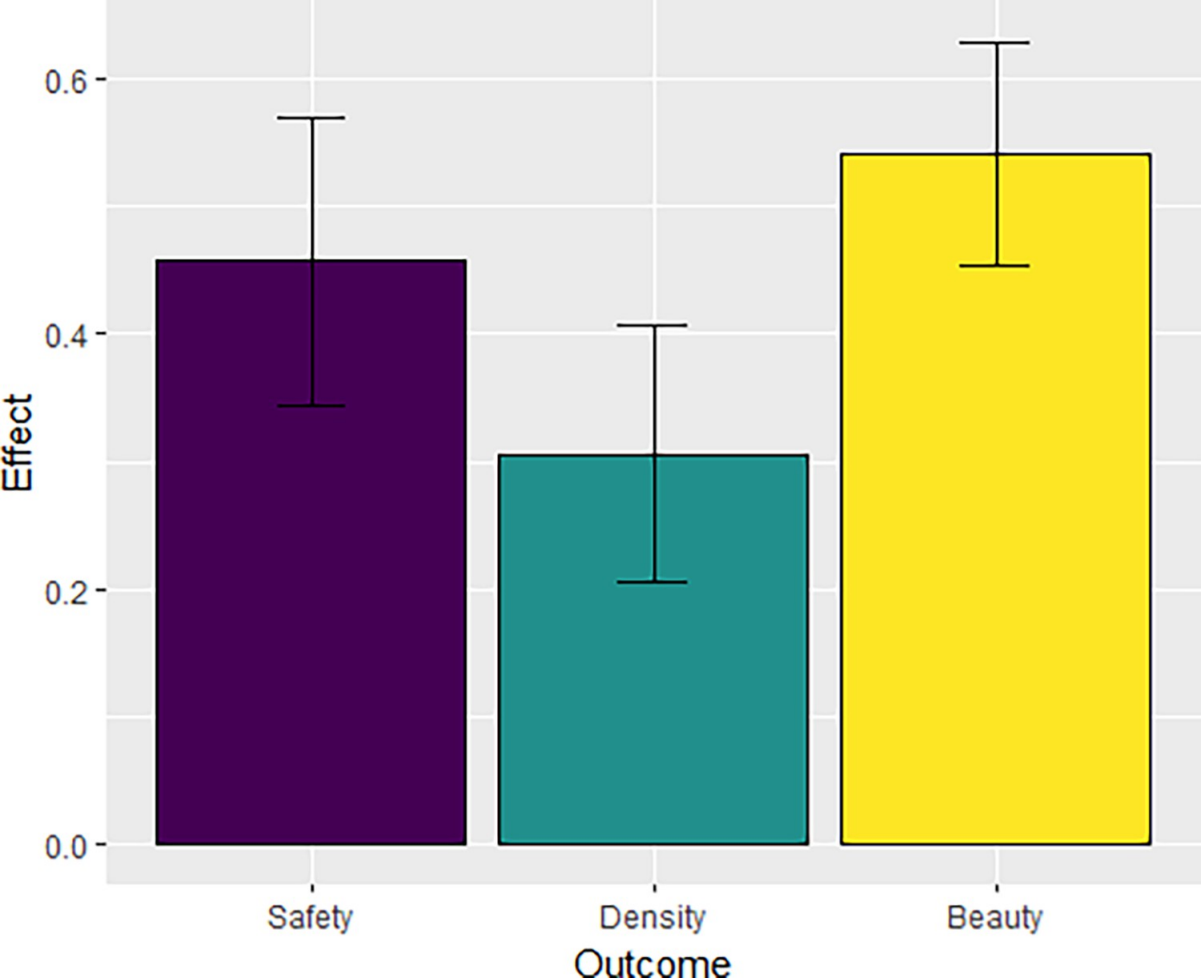
The significant effects of perceived greenness on other outcome variables, when controlling for years lived experience in urban environments and presence and number of trees.

In fact, beauty is impacted significantly by perceived greenness even when controlling for objective number of trees, results of a linear prediction model fitted against perceived greenness and perceived beauty with controls for number of trees and years lived in urban areas can be seen in Table 5. The full effect of perceived greenness on outcome variables safety, beauty, and density is presented in Fig 10.

**Table 5:** Results of linear model on perceived beauty against perceived greenness.

Variable	Estimate	p-Value
Intercept	1.82	0.0101
Greenness	0.54	1.63e-08
*SomeTrees*	0.61	0.0368
*MoreTrees*	0.80	0.0214

**Fig 11:**
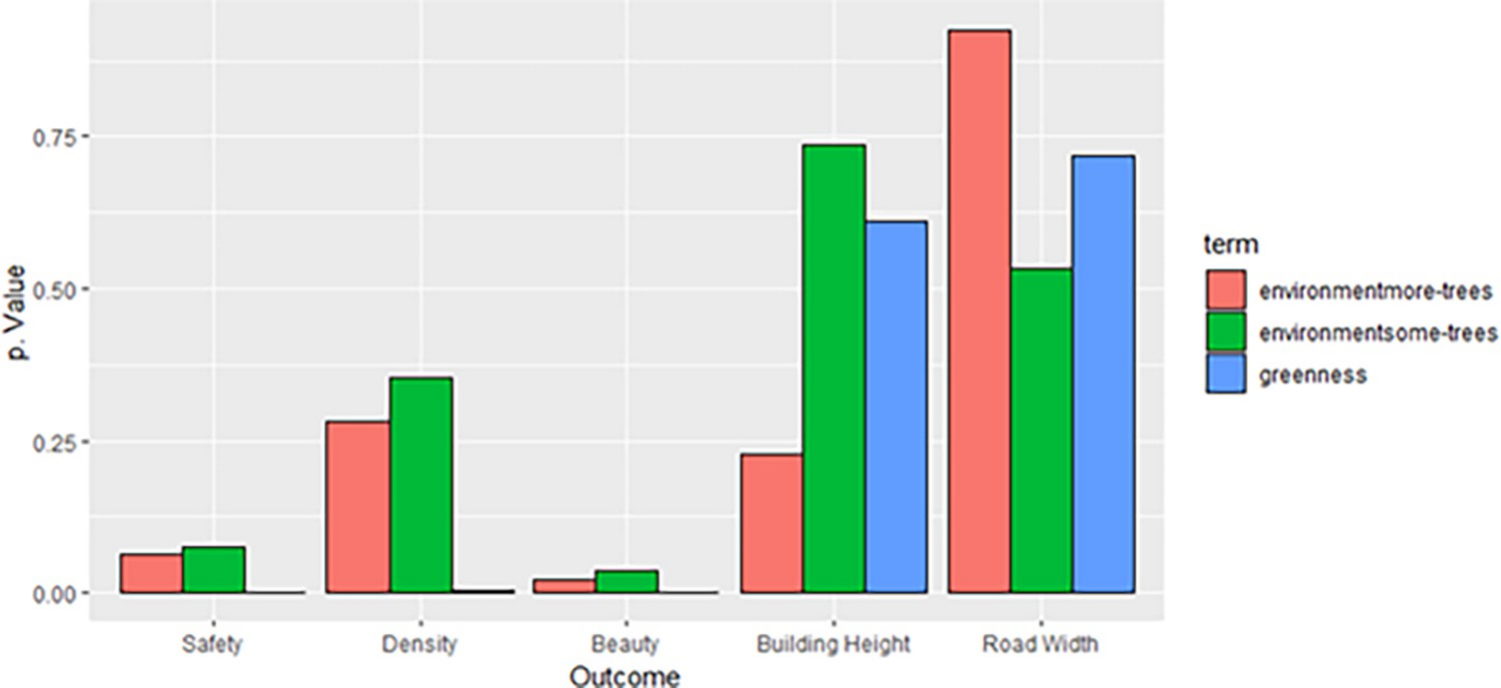
p-value comparison for linear models fitted against each of the outcome variables, for number of trees compared to perceived greenness.

We can further evaluate the importance and significance of perceived greenness as a predictor by comparing the p-values of linear models fitted for perceived greenness against each of the other outcome variables, compared to those for number of trees against outcomes. Fig 11 shows that for safety, density, and beauty, perceived greenness is the most significant predictor when controlling for lived urban experience and environment. For building heights, abundance of trees is the most significant predictor, as is presence of trees for road width. Note, though, that all predictors for building height and road width have a p-value greater than 0.05, as do environmental predictors for safety and density.

Finally, random forest prediction models were generated to explore variable importance for predicting each of the outcomes. Table 6 shows each outcome’s most important variable that is not in itself an outcome variable. Table 7 shows the most important variables including other outcome variables, to help identify correlations within the outcomes themselves.

**Table 6:** Variable importance excluding outcome variables as predictors.

Outcome	Predictor	Increase in Node Purity
Safety	Years Urban	16.75
Density	Years Suburban	8.20
Beauty	Years Suburban	16.08
Greenness	Years Urban	7.28
Building Height	Years Suburban	5.43
Road Width	Years Suburban	8.69

**Table 7:** Variable importance including the outcome variables as predictors.

Outcome	Predictor	Increase in Node Purity
Safety	Beauty	68.69
Density	Building Height	25.87
Beauty	Greenness	133.57
Greenness	Beauty	153.33
Building Height	Road Width	28.73
Road Width	Building Height	33.13

## Discussion and conclusions

This study has aimed to use IVE technology to explore the impact of added greenery on perceptions of an urban space. Our findings show that the inclusion of trees greatly increased ratings of greenness and beauty in the virtual environment, and very slightly increased ratings of density and building heights, two factors associated with negative elements of urban environments.

Findings also show a strong relationship between years lived in urban areas and ratings of safety, building heights and road width. 6+ years lived in urban areas significantly increase perceived safety, implying those with less urban experience may find the urban environment less safe. <1 year urban experience significantly increased perceived building height, but 11+ years also increased it. 6–20 years suburban experience significantly reduced perceived road width, as did 11–20 years urban. The inconsistencies in these results may be a product of the unrepresentative sample. The findings do suggest that individual experience in urban environments may impact what constitutes a ‘4 out of 7’ road width, implying that individual contexts and backgrounds are a large factor of environment perception.

When controlling for the effect of years lived in urban areas, the impact of added trees was lessened considerably, leaving perceived beauty as the only outcome variable still significantly impacted by trees alone. However, when examining the effect of perceived greenness—meaning the impact of the rating of greenness on the other outcome variables—beauty, density, and safety were all significantly predicted, even when accounting for years lived in urban areas. While perceived greenness is strongly impacted by the presence and abundance of trees, its relationship to beauty is significant even when number of trees is controlled for.

The virtual environment was successful in reducing time-cost and ease of development, as it required only a basic understanding of CityEngine and Unity to develop. However, the money-cost of VE development would be considered high. While Unity is free, CityEngine is proprietary software that not all researchers could afford.

Further evaluation is required on the system’s Level of Immersion, but it can be roughly assessed against [92]‘s six-point framework, for which it comes out reasonably inclusive (1832x1920 resolution per eye, 120Hz refresh rate, no lag, no cables, light headset), surrounding (wide field of view, accommodation for depth perception), and matching (head-tracking, physical walking movement available), though its vividness and plot could be considered lacking (no intentional plot included, shadows but no dynamic objects, high resolution but no subjective ratings of realism taken).

This paper’s findings present two opportunities for further exploration in the perceptions of urban space, the first being the isolation of perceived greenness as a factor of urban perceptions, either wholly or partly independent of objective greenness. The second is the use of IVEs for investigation of urban perceptions, with limited hardware and minimal time-cost, that can be easily reproduced as a testing tool for future study.

Further research could focus more directly on the effects of lived experience in different levels of urbanisation on perceptions of the urban environment, by including in its sample more participants with rural backgrounds. Future iterations of this study should include more robust questionnaire design, with definitions of terms and examples of the minimum and maximum ratings in each category.

This paper concludes that, while further study is needed, both the introduction and increase in quantity of urban road-side greenery will impact perceptions of beauty in an urban environment, but that the perception of greenness is in itself a separate predictor for perceived beauty.
