# Correction: Evaluating the impact of added greenery on perceived factors of an urban environment in virtual reality

**DOI:** 10.1371/journal.pone.0321871

**Published:** 2025-04-02

**Authors:** Ron Bar-Ad, Markel Vigo, Geoffrey Caruso, Qudamah Quboa, Nuno Pinto

The images for Figures 1-5 are out of order. The figure captions appear in the correct order. Please see the correct Figures 1-5 here.

**Fig 1:**
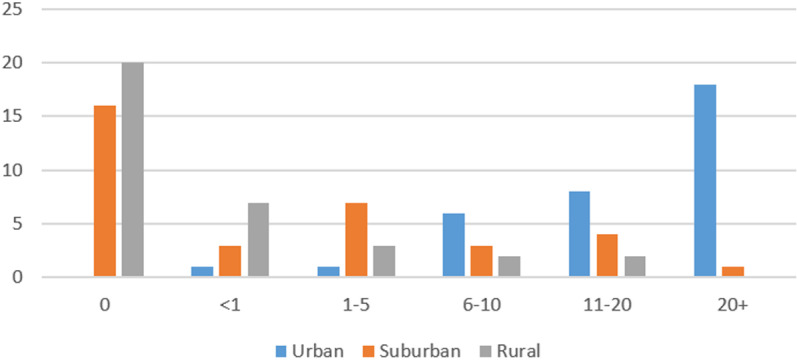
Sample lived experience per environment.

**Fig 2:**
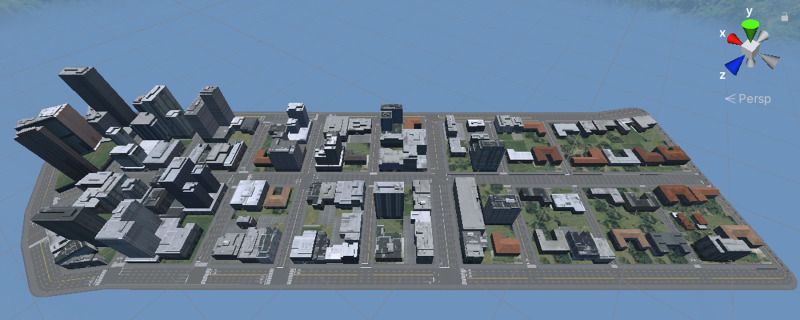
A bird’s-eye-view of the VE with no trees.

**Fig 3:**
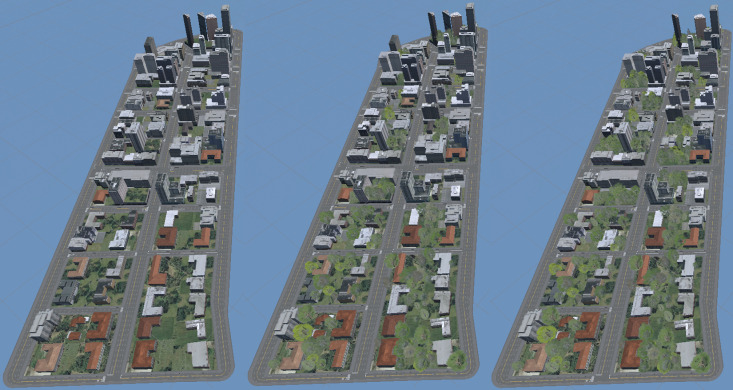
Environments (from left to right) NoTrees, SomeTrees and MoreTrees, side by side.

**Fig 4:**
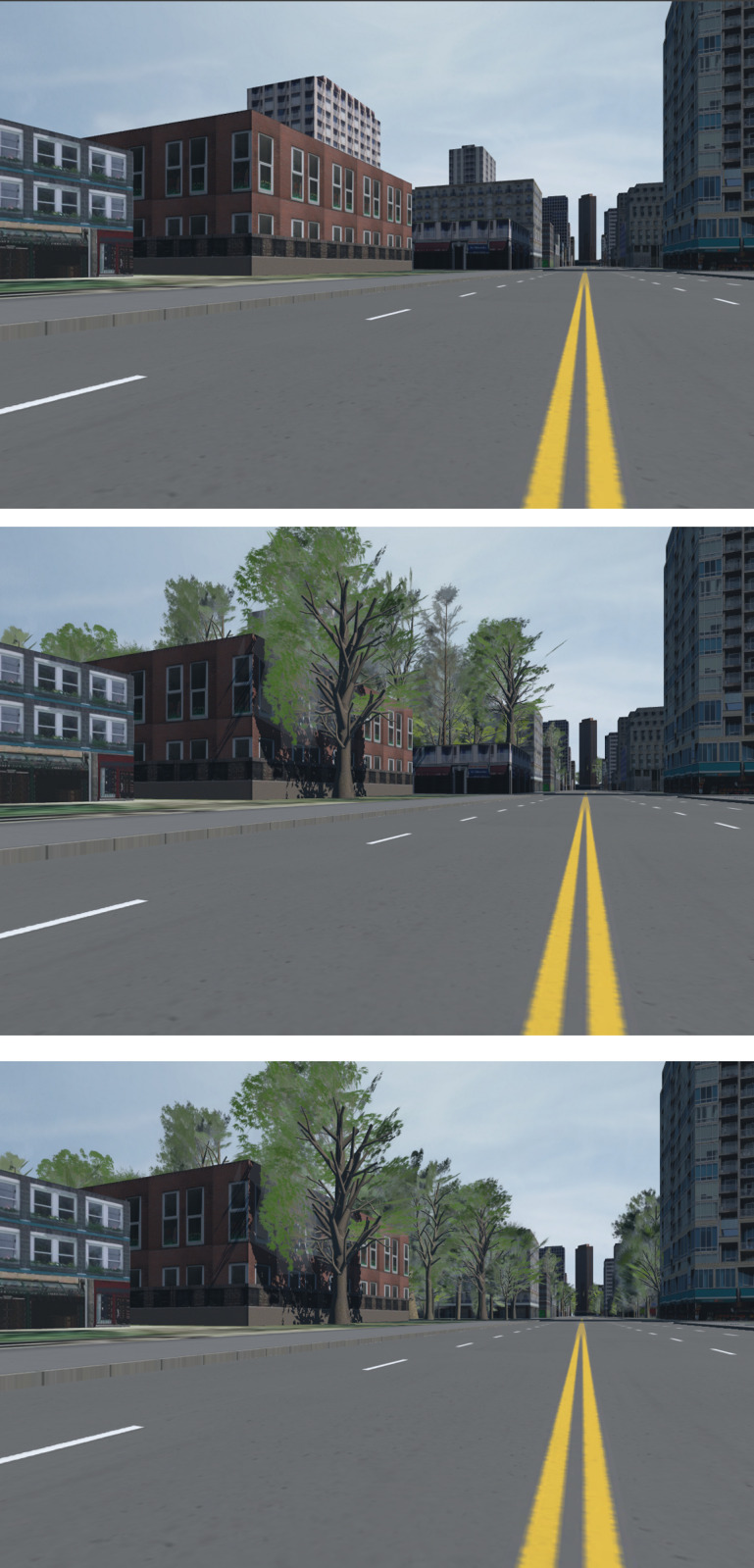
Environments (from top to bottom) NoTrees, SomeTrees and MoreTrees, from a street-level perspective.

**Fig 5:**
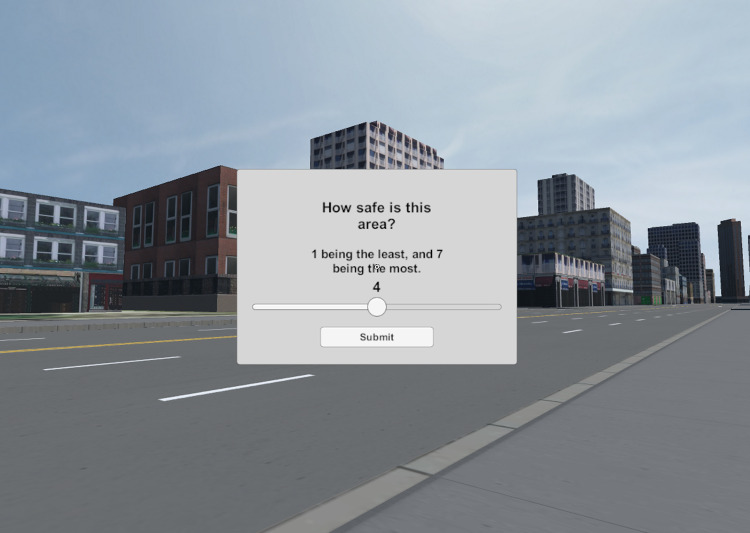
A view of the NoTrees environment with the questionnaire up.
